# Cardiac Endocrine Function and Hormonal Interplay in Pediatrics: From Development to Clinical Implications

**DOI:** 10.3390/biomedicines13092225

**Published:** 2025-09-10

**Authors:** Valeria Calcaterra, Savina Mannarino, Filippo Puricelli, Giulia Fini, Giulia Cecconi, Martina Evangelista, Beatrice Baj, Cassandra Gazzola, Gianvincenzo Zuccotti

**Affiliations:** 1 Department of Internal Medicine and Therapeutics, University of Pavia, 27100 Pavia, Italy; 2 Pediatric Department, Pediatric Unit, Buzzi Children’s Hospital, 20154 Milano, Italy; cassandra.gazzola@unimi.it (C.G.); gianvincenzo.zuccotti@unimi.it (G.Z.); 3 Pediatric Department, Pediatric Cardiology Unit, Buzzi Children’s Hospital, 20154 Milan, Italy; savina.mannarino@asst-fbf-sacco.it (S.M.); filippo.puricelli@asst-fbf-sacco.it (F.P.); giulia.fini@asst-fbf-sacco.it (G.F.); giulia.cecconi@asst-fbf-sacco.it (G.C.); martina.evangelista@asst-fbf-sacco.it (M.E.); beatrice.baj@unimi.it (B.B.); 4 Department of Biomedical and Clinical Science, University of Milano, 20157 Milano, Italy

**Keywords:** heart, cardiac, endocrine system, hormones, pediatrics, children

## Abstract

The endocrine system plays a pivotal role in all stages of cardiac development and in maintaining the structural and functional integrity of the heart. Notably, the heart itself functions as an endocrine organ, producing hormones that regulate blood pressure, fluid balance, and myocardial remodeling. This narrative review explores the endocrine mechanisms underlying cardiac development and function, with a focus on fetal and pediatric life. Special attention is given to the heart’s intrinsic endocrine activity and how hormonal signals interact with the cardiovascular system during early development. Hormonal signaling is essential for maintaining physiological homeostasis and supporting proper heart development during growth. Disruptions in these signals may serve as silent precursors to structural or functional heart disease, potentially manifesting later in life. Understanding these interactions is clinically relevant, as endocrine imbalances can contribute to the onset, progression, and prognosis of pediatric cardiac disorders. Early identification of hormonal dysregulation can help prevent or mitigate adverse cardiovascular outcomes. Furthermore, recognizing age-specific patterns in hormone–heart interactions may enable the development of targeted diagnostic and therapeutic strategies.

## 1. Introduction

The heart is the first organ to form and function during embryonic development, and its early maturation is essential for the viability of the growing embryo [1]. Cardiac development begins as early as the third week of gestation, guided by a complex interplay of molecular pathways, transcription factors, and morphogenetic cues that drive the differentiation and organization of cardiogenic progenitor cells into a structurally and functionally competent heart [2,3]. This process culminates in the formation of a four-chambered heart capable of supporting the fetal circulatory needs and preparing for the dramatic hemodynamic changes that occur at birth.

However, beyond the intrinsic genetic cardiogenesis programs, the endocrine system plays a fundamental and often underappreciated role in modulating every phase of cardiac development. Hormones such as thyroid hormones (THs) cortisol, insulin-like growth factors (IGFs), insulin, and sex steroids are key modulators of cardiac cell proliferation, metabolic reprogramming, sarcomere maturation, and mitochondrial biogenesis [4,5,6,7]. These hormones not only act systemically but also exert local paracrine effects within the fetal heart, creating a tightly regulated hormonal microenvironment that adapts dynamically throughout gestation.

For instance, THs are critical in promoting terminal differentiation of cardiomyocytes and the postnatal metabolic switch from glycolysis to oxidative phosphorylation [8]. Glucocorticoids facilitate structural remodeling and upregulate genes involved in calcium handling and contractile function [9]. Meanwhile, IGF-II and insulin drive early myocardial growth through proliferation, while IGF-I becomes more prominent in late gestation, supporting hypertrophy and maturation [10,11]. These processes are not isolated; rather, they are temporally coordinated through hormonal surges and feedback mechanisms involving the hypothalamic–pituitary–thyroid (HPT), hypothalamic–pituitary–adrenal (HPA), and pancreatic axes.

Disruption of this finely tuned endocrine environment, whether due to maternal disease, intrauterine stress, genetic syndromes, or iatrogenic factors, can significantly impair fetal cardiac development [12]. Emerging evidence links prenatal hormonal imbalances to congenital heart defects (CHDs), altered cardiomyocyte function, and long-term cardiovascular dysfunctions such as hypertension, metabolic syndrome, and arrhythmias [13,14].

Importantly, the heart itself acts as an endocrine organ, particularly through the production of atrial and brain natriuretic peptides (ANP and BNP), which regulate blood pressure, fluid homeostasis, and myocardial remodeling. These cardiac hormones have gained clinical significance in pediatric cardiology, not only as markers of heart failure but also as tools to assess disease severity, guide therapy, and predict outcomes in children with CHDs, cardiomyopathies, pulmonary hypertension, and inflammatory syndromes such as Kawasaki disease and multisystem inflammatory syndrome in children (MIS-C) [15,16,17,18].

Given the complex, bidirectional relationship between the endocrine and cardiovascular systems, understanding the hormonal regulation of cardiac physiology is crucial for both developmental biology and clinical practice. Hormonal signaling pathways not only shape cardiac architecture during development but also influence the heart’s capacity to adapt, repair, and respond to physiological or pathological stress across the lifespan [6].

The aim of this narrative review is to explore the endocrine mechanisms involved in cardiac development and function, including the heart’s intrinsic endocrine activity, with particular focus on how hormones interact with the cardiovascular system during fetal life and childhood. We also investigate the clinical relevance of these interactions in pediatric pathologies, highlighting how endocrine imbalances may contribute to disease onset, progression, and prognosis. This review integrates developmental endocrinology and pediatric cardiology to provide a comprehensive perspective on the hormone–heart relationship and its relevance to diagnosis, treatment, and preventive care.

## 2. Methods

A narrative review was conducted to explore the endocrine regulation of cardiac development and function, including the heart’s own endocrine activity, with particular emphasis on hormonal interplay during fetal life and childhood, and its clinical implications in pediatrics.

The literature search was performed using PubMed and Scopus databases, covering publications from the past 10 years (2015–2025). The keywords used, either individually or in combination, included: hormones, endocrine system, endocrine function, heart, development, atrial natriuretic peptides, brain natriuretic peptides, heart function, children, pediatrics, congenital heart diseases.

Inclusion criteria comprised original research articles, clinical trials, meta-analyses, reviews, and cases series published in English within the last decade. Studies primarily focusing on pediatric populations were prioritized; however, research involving adult populations or animal models was also included if it contributed to understanding the relevant mechanisms or offered translational insights into pediatric conditions.

Exclusion criteria included editorials, commentaries, letters, case reports, and all articles not published in English.

The initial search identified 990 articles. Of these, 185 were excluded prior to screening due to duplication or other reasons, leaving 805 articles for screening. After reviewing titles and abstracts, 185 full-text articles were assessed for eligibility, and 130 met the inclusion criteria for the review. The reference lists of the included studies were also examined to identify any additional relevant publications.

**Figure 1:**
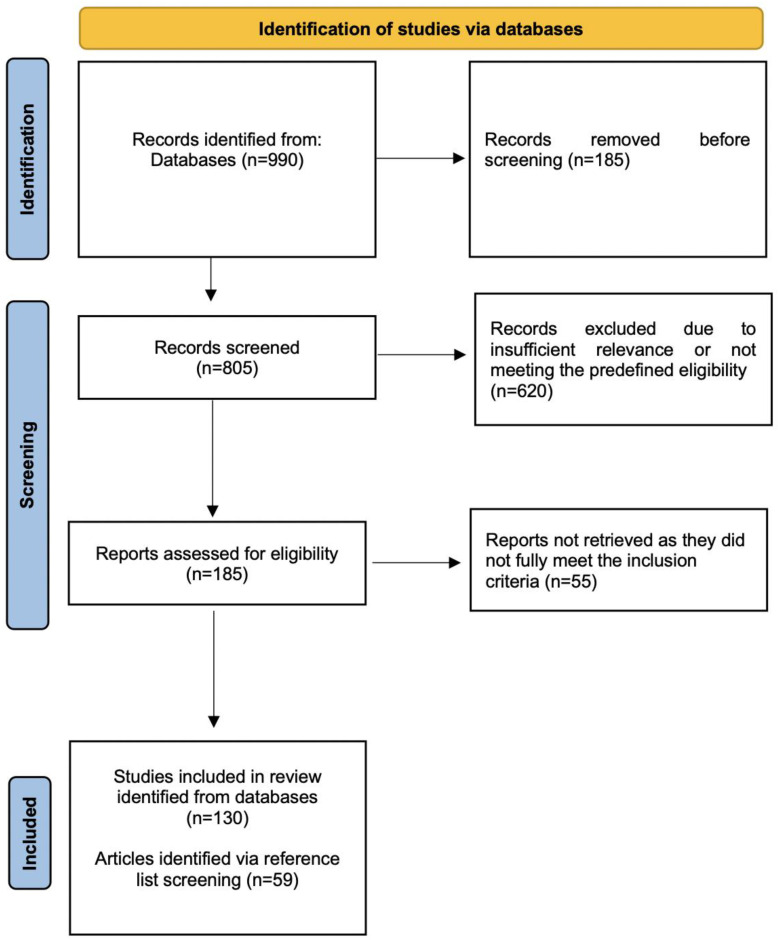
Flowchart of article selection.

The article selection process is summarized in Figure 1.

## 3. Fetal Heart Development

The heart is the first functional organ to complete its formation developing from a group of specialized progenitor cells through the activation of specific cellular and molecular signals.

Heart formation begins during gastrulation (day 15–17 postconception), when mesodermal progenitors migrate from the primitive streak to the antero-lateral plate mesoderm [19,20]. These progenitors diversify into the first heart field (FHF) and second heart field (SHF). The FHF primarily generates the linear heart tube, from which the left ventricle and parts of the atria will develop, whereas the SHF is a source of cells that add to the poles of the heart tube contributing in the development of the right ventricle and outflow tract at the arterial pole of the heart and to part of the atria at the venous pole. Expression of specific transcription factors (such as NKX2-5, GATA4, ISL1, TBX5, and MEF2C) drives commitment and differentiation of cardiac lineage cells [21]. At around 19 days of gestation FHF-derived bilateral endocardial tubes fuse at the midline to form the primitive heart tube. This tube, comprising an inner endocardium, cardiac jelly, and outer myocardium, begins contracting around day 21 of gestational age [19,20]. By approximately day 22–28, rightward looping (also known as D-looping) occurs, transforming the linear structure into a curved configuration allowing the alignment of the atrio-ventricular canal and of the outflow tract for subsequent septation. At about 7 weeks of gestation the heart achieves a four-chambered structure with distinct systemic and pulmonary outlets. The intracardiac septations of the atria, the ventricles, and the outflow tracts are fundamental to complete heart formation and they all are interdependent [19,20]. The cardiac conduction system arises from specialized cardiomyocyte populations: the sinoatrial node originates from the posterior SHF and the atrioventricular node with the His–Purkinje system originates from the atrioventricular canal [22].

After birth, environmental conditions and demands on the heart change dramatically. Postnatally, cardiomyocytes are exposed to higher oxygen levels and must cope with increased blood pressure and mechanical load. Consequently, extensive changes in the myocardium occur during late gestation and the postnatal period in order to adapt to these new demands. During cardiac maturation, the vast majority of cardiomyocytes exit the cell cycle, losing the ability to reproduce and regenerate, and initiate essential metabolic, structural, and electrophysiological changes [23]. The major metabolic change is the shift from a metabolism based on glycolysis during fetal life to the more efficient oxidative phosphorylation. At the same time, there is also an increase in the number, size, and complexity of mitochondria [23]. Structural changes include binucleation and polyploidization, reorganization and hypertrophy of the sarcomeres, development of T-tubules, accumulation of gap junctions at the intercalated discs, and switching to mature isoforms of structural proteins and membrane channels that improve the electromechanical performance of the cardiomyocyte [23]. All these processes appear to be driven by endocrine signals related to normal fetal development and postnatal adaptation.

## 4. Hormonal Regulation of Heart Development and Function

### 4.1. Hormonal Control of Heart Development in Fetal Life

Hormonal signals have a crucial role in cardiomyocyte proliferation, maturation, and metabolic programming during fetal development and postnatal life. TH, IGFs, GH, insulin, and adrenal hormones each play critical and distinct roles in shaping the embryonic heart. This complex hormonal interplay ensures the heart develops in a way that supports both feto-maternal circulation and the transition to an independent circulation after birth. In the next subsections we will display how each hormone impacts on fetal heart development.

#### 4.1.1. Thyroid Hormones

During pregnancy and embryogenesis, thyroxine (T4) and triiodothyronine (T3) supply is entirely provided by maternal sources. These hormones are essential for proper organogenesis to occur, including cardiac embryogenesis [20,23,24]. From the 28th gestational week until term, there is a transition from complete maternal dependence to autonomous fetal synthesis of these hormones. This transition is granted and triggered by a physiological increase in fetal cortisol levels, which upregulates deiodinase enzymes, key regulators of thyroid hormone metabolism. The deiodinase family, consisting of three isoenzymes: D1, D2 and D3, regulate local activation or inactivation of thyroid hormones in a tissue-dependent manner. Specifically, D1 is active in the liver, kidney, thyroid, and pituitary gland and converts T4 to T3 and reverse T3 (rT3) whereas D3, expressed in the placenta and fetal tissues, inactivates T3 and converts T4 to rT3, thus protecting the fetus from hormone excess. During pregnancy, D3 predominates; near term the increase in cortisol reduces D3 activity in favor of D1; this switch is necessary to allow the fetus to meet postnatal metabolic demands through the autonomous production of thyroid hormones [24].

The hypothalamus–pituitary–thyroid gland axis governs TH production. Because of endogenous and environmental cues, the hypothalamus secretes thyrotropin-releasing hormone (TRH), which will activate the pituitary gland and it will release into the circulation thyroid-stimulating hormone (TSH). Finally, the thyroid-stimulating hormone stimulates follicular cells of the thyroid gland to synthesize the prohormone T4 and the activated form T3. Upon binding of T3, nuclear thyroid receptors (TRα and TRβ) form heterodimers with the retinoic acid receptor and interact with the T3 response elements (TREs) to activate gene expression. Moreover, THs can bind cell surface receptors and mediate non-genomic pathways, such as the phosphatidylinositol 3-kinase (PI3K)/protein kinase B (AKT)/mTOR pathway and mitogen-activated protein kinase (MAPK)/extracellular signal-regulated kinase (ERK). Both signaling pathways are involved in controlling cell cycle progression and proliferation [5].

Thyroid hormones surge near term, triggering cardiomyocyte maturation, indeed T_3_ engaging MAPK/PI3K pathways promotes structural and metabolic transitions within cardiomyocytes [5]. Specifically, T_3_ downregulates cell cycle genes, reducing proliferation, and contributes to sarcomere maturation, improved calcium handling and enhanced mitochondrial biogenesis. In collaboration with cortisol, T_3_ also helps the transition from IGF-II to IGF-I expression patterns, serving as a maturational switch [25].

There is increasing interest in studying how thyroid hormones interact with the cardiovascular system, especially in light of the hormones’ possible ability to regenerate the myocardium. It has been demonstrated that T3 promotes the proliferation of cardiomyocytes, particularly during the early phases of development. Exogenous T3 administration seems to activate the ERK1/2 signaling pathway via IGF-1R, promoting cell proliferation in response to injury or trophic stimuli in neonatal mouse models, where cardiomyocytes retain immature characteristics and proliferative capacity [26].

This regenerative capacity, however, appeared to decrease after the sixth postnatal day; beyond this threshold, cardiomyocytes appeared to be refractory to mitogenic stimuli. Further investigations in the area of regional myocardial proliferation, conducted by Bogush et al. [10], showed that administration of T3 (3.5 ng/g/day for three consecutive days, from postnatal day 7 to day 9) induces a significant increase (of about 18%) in the number of cardiomyocytes in the left ventricle, with proliferation predominantly localized to the ventricular apex. The phosphatase DUSP5, which prevents ERK1/2 activation, controls this phenomenon. From the ventricular base to the apex, DUSP5 expression progressively rises during development, progressively restricting the proliferation of cardiomyocytes. The regional specificity of the T3-induced proliferative response at the apex was confirmed by clonal lineage tracing. Subsequent research revealed that T3 was able to restore cardiomyocyte proliferation through genetic inhibition of DUSP5, supporting the previously stated thesis and suggesting possible therapeutic uses for myocardial regeneration following infarction [27]. The idea that thyroid hormones may influence cardiac regeneration during neonatal life and possibly beyond is supported by these findings, which are consistent with larger studies investigating the activation of the ERBB2 pathway, which is linked to cardiomyocyte dedifferentiation and proliferation [28,29].

In addition to its proliferative and regenerative effects, thyroid hormones contribute significantly to postnatal cardiac maturation by promoting mitochondrial biogenesis and facilitating the metabolic switch to oxidative phosphorylation. T3 increases the number and density of mitochondrial cristae within cardiomyocytes. This increase is necessary to meet the increased energy requirements for cardiac growth and functional efficiency [30].

#### 4.1.2. Corticosteroid Hormones

Corticosteroid hormones (CHs) include mineralocorticoids and glucocorticoids. The pituitary gland releases adrenocorticotropic hormone (ACTH) which induces the secretion of mineralocorticoids and glucocorticoids in the cortex of the adrenal glands.

Cortisol is the predominant glucocorticoid in humans. Upon stimulation by ACTH or angiotensin II, cortisol is synthesized in the mitochondria via conversion of cortisone by type 1 11β-hydroxysteroid dehydrogenase.

Cortisol can bind to the glucocorticoid receptor (GR) and the closely related mineralocorticoid receptor (MR). The GR is represented in most tissues by two main isoforms (GRα and GRβ) [23,31]. Ligand binding triggers homodimerization and translocation to the nucleus, where the GR interacts with DNA response elements (GREs) to activate the genomic pathway [32]. GRs can also bind to the circular DNA of mitochondria, thereby regulating mitochondrial gene transcription. Cortisol can also impact gene expression via the interaction of the GR with other transcription factors, such as nuclear factor-κB (NF-κB) and activator protein-1 (AP-1) [4]. Membrane-associated GRs can also mediate non-genomic pathways such as PI3K/MAPK signaling, PLC signaling, and the Ca^2+^/calmodulin-dependent protein kinase II pathway, promoting cardiomyocyte maturation [33].

The prepartum cortisol surge contributes to initiating structural, functional, and metabolic changes in the fetal myocardium, synergically with THs and IFG-I. In fact, cortisol stimulates local T3 production in the cardiac tissue and collaborates in the IGF-II to IGF-I switch at late gestation by downregulation of IGF-II [25].

The principal mineralocorticoids are aldosterone and deoxycorticosterone, secreted by the zona glomerulosa of the adrenal cortex under the regulation of ACTH, plasma potassium, and the renin–angiotensin–aldosterone system (RAAS) [34]. Aldosterone binds to the MR, classically regulating sodium reabsorption, potassium excretion, and extracellular fluid volume [35]. In the fetus, these actions are critical for maintaining blood pressure stability and optimizing placental perfusion. By influencing vascular resistance and intravascular volume, mineralocorticoids indirectly affect cardiac preload and systemic hemodynamics [36]. Importantly, MRs are expressed in the developing myocardium and vasculature. Their activation contributes to vascular remodeling and may influence cardiomyocyte growth [35].

Deoxycorticosterone, though less potent, appears to provide redundancy during fetal life, ensuring vascular tone and electrolyte balance [37].

#### 4.1.3. Catecholamines

The adrenal medulla secretes epinephrine and norepinephrine, whose concentrations increase during gestation and peak in the perinatal period [38]. This surge is essential for the dramatic cardiovascular adjustments at birth.

Catecholamines act primarily through β-adrenergic receptors, enhancing myocardial contractility, heart rate, and stroke volume, thereby ensuring adequate cardiac output under intrauterine stress conditions such as hypoxia or labor [39]. Through α-adrenergic receptors, they modulate vascular tone and systemic blood pressure, contributing to redistribution of blood flow towards vital organs such as the brain, heart, and adrenal glands during hypoxic episodes [40].

Beyond these immediate effects, catecholamines influence cardiac gene expression and structural remodeling, including hypertrophic growth and metabolic flexibility [41]. Their role in stress responsiveness during fetal life is crucial for survival at birth, where the sudden transition from placental to pulmonary circulation requires rapid cardiovascular adaptation [42].

Inadequate catecholamine signaling can impair neonatal blood pressure regulation, contractility, and thermogenesis, highlighting their essential role in perinatal cardiovascular resilience [43].

#### 4.1.4. Sex Hormones

SHs are mainly secreted by the testes and the ovaries, where they play a primary role in sexual differentiation and reproductive development. However, a fraction of SHs, especially adrenal androgens, is also synthesized in the adrenal cortex (zona reticularis)**,** representing a minor but functionally relevant contribution during fetal life.

Among other hormones, the anterior pituitary secretes gonadotropins, which regulate the production of estrogens from the ovaries in the female and androgens from the testes in the male. In postmenopausal women and men, estrogens are converted predominantly from adrenal and ovarian or testicular androgens. Sex-hormone-binding globulin (SHBG) regulates the levels of free sex hormones that can readily enter the cell by passive diffusion, but it has also been shown that cellular uptake of SHBG-bound androgens and estrogens could be realized by endocytosis and that sex steroid signaling, at least in part, depends on this route [44].

Estrogens can bind to three forms of estrogen receptors (ERα, ERβ, and G-protein-coupled estrogen receptor (GPER)). In the nucleus, estrogen binding to ERs triggers the recruitment of coactivators that facilitate binding to DNA via estrogen response elements (EREs), mediating the effects of estrogens via the genomic pathway [44]. ERs can also bind to mitochondrial DNA and induce transcription of mitochondrially encoded genes directly or indirectly via nuclear respiratory factor-1 (NRF-1) [18]. ERs located in the plasma membrane or cytosol can mediate more rapid effects via non-genomic pathways, such as PI3K/AKT/mTOR and MAPK/ERK [44].

In the same way as estrogens, androgens cross the cell membrane and primarily bind to nuclear androgen receptors (ARα and ARβ), thereby inducing the genomic pathway via DNA response elements in target gene promoters. Testosterone can be converted intracellularly to dihydrotestosterone, which forms much more stable complexes with ARs and thus can amplify the androgen signaling in individual cells [23]. Membrane-bound receptors mediate non-genomic effects most prominently via the activation of protein kinase A (PKA) and protein kinase C (PKC) but also via activation of MAPK/ERK [23].

Besides well-known cardioprotective effects of estrogens during adult life, some studies also hint at the impact of sex hormones on the electrophysiological maturation of cardiomyocytes [23,45]. Estrogens also induce the transcription of mitochondria-encoded genes relevant for oxidative phosphorylation, enforcing the metabolic switch during cardiomyocyte maturation [33]. While the major hallmarks of cardiomyocyte maturation apply to both sexes, mitochondria demonstrate a clear sexual dimorphism, which is thought to originate from the exclusive maternal inheritance of their genome, resulting in an optimized function in female compared to male individuals [33]. This seems to contribute to gender differences in cardiac function, as well-summarized by Ventura-Clapier et al. [33].

#### 4.1.5. Growth Hormone/Insulin-like Growth Factors and Insulin

GH exerts significant effects on cardiac growth, function, and contractility. Many of these actions are mediated through IGF-I, which is primarily produced in the liver in response to GH stimulation. Therefore, the GH/IGF axis represents a central regulatory pathway in cardiac development and adaptation.

IGFs and insulin have an important role in the regulation of intrauterine growth [25,46]. IGF-I is primarily produced in the liver in response to GH, but the bioavailability of IGF-I/-II is regulated by their receptors (IGF-R), by the IGF-binding proteins, and by themselves. IGFs can bind either to their receptors or to the insulin receptor (IR) due to some structural homology. IGF-IRs belong to the receptor tyrosine kinase family and triggers phosphorylation of intracellular lipids, second messengers, and serine/threonine kinases upon ligand binding. Thereby, they activate non-genomic pathways such as PI3K/AKT/mTOR and MAPK/ERK [23,25]. IGF-I can also activate phospholipase C (PLC) via a heterotrimeric G protein, initiating an inositol 1,4,5-trisphosphate (IP3)-dependent signaling pathway that eventually affects intracellular calcium levels [23]. IGF-II can bind to IGF-IR but also to the mannose 6-phosphate receptor with high affinity, which is, therefore, known as the IGF-IIR. The IGF-IIR lacks the tyrosine kinase domain, therefore, binding this receptor results in internalization and degradation of the ligand instead of inducing phosphorylation cascade [23]. Accordingly, the bind of IGF-II to the IGF-IIR inhibits the signaling of IGF-II via the IGF-IR. Therefore, the main role of the IGF-IIR is to regulate IGF signaling.

IGF-II is the primary in utero growth factor, essential for cardiac mass expansion via cardiomyocyte proliferation. Near term, GH receptor expression increases, inducing IGF-I synthesis as IGF-II declines [25]. IGF-I promotes fetal cardiomyocyte hypertrophy and maturation, synergistically with THs and cortisol [5]. Insulin supports myocardial growth alongside and enhancing glucose transport and metabolic efficiency. Unlike IGF-I, insulin primarily stimulates mass growth rather than differentiation [25].

Beyond these indirect mechanisms, GH also exerts direct effects on the heart. GH receptors are expressed in cardiomyocytes, and their activation influences cardiac mass, geometry, and performance. Experimental and clinical studies have shown that GH can enhance myocardial contractility, improve diastolic function, and modulate vascular resistance [47,48]. Thus, the GH/IGF axis integrates both endocrine and paracrine/autocrine mechanisms, representing a central pathway in cardiac development and adaptation.

**Figure 2:**
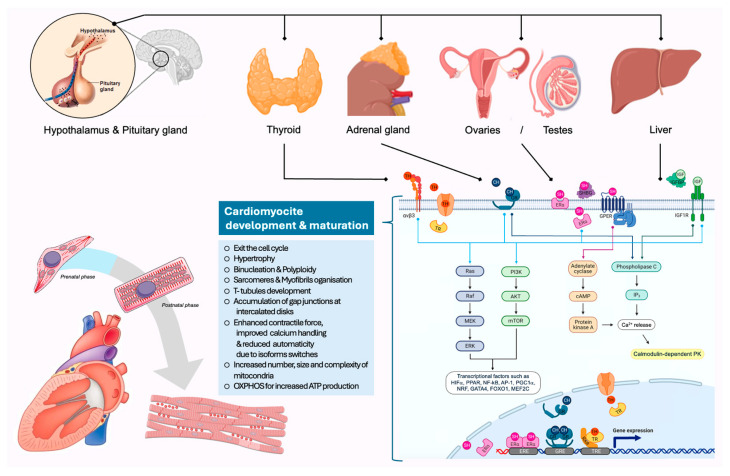
Endocrine regulation of fetal heart development. Various hormones contribute to the growth, maturation, and differentiation of cardiomyocytes during fetal development. Key hormonal players include thyroid hormones, adrenal and sex hormones, insulin-like growth factors, catecholamines, and natriuretic peptides. These hormones act through specific molecular signals and transduction pathways, modulating processes such as proliferation, hypertrophy, mitochondrial maturation, and cardiac energy metabolism. The coordinated interaction of these signals is essential to ensure the formation of a functionally competent heart at birth.

The endocrine regulation of fetal heart development is schematically represented in Figure 2.

Table 1 provides an overview of the major hormones implicated in cardiovascular development and function during fetal life.

### 4.2. Hormonal Effects on Heart Function in Childhood

#### 4.2.1. Cardiac Effects of Thyroid Hormones

The thyroid mainly produces T3 and T4. Most circulating hormone is T4, an inactive prohormone, which is peripherally converted into the active T3. T3 acts by binding to nuclear receptors, stimulating the transcription of genes encoding contractile proteins (e.g., myosin isoforms) and proteins involved in intracellular calcium regulation (e.g., SERCA2 and phospholamban) [49]. In addition to genomic effects, thyroid hormones exert rapid non-genomic actions: they modulate membrane ion channels (Na^+^, K^+^, Ca^2+^), influencing cardiac excitability and contractility. Moreover, thyroid hormones activate intracellular signaling pathways such as those mediated by phosphatidylinositol 3-kinase (PI3K) and protein kinase B (AKT); these signaling pathways enhance cell survival and physiological myocardial hypertrophy. Through the previously described mechanisms, which are activated with distinct temporal patterns, thyroid hormones exert an overall stimulatory effect on the cardiovascular system. This effect is manifested by positive chronotropic and inotropic actions, an increase in cardiac output, peripheral vasodilation, enhanced cellular metabolic activity, and elevated oxygen consumption [49]. In addition, THs improve diastolic relaxation (positive lusitropic effect) and increase cardiac preload through activation of the renin–angiotensin–aldosterone system. They also reduce systemic vascular resistance via direct vasodilatory actions on vascular smooth muscle. At the cellular level, thyroid hormones upregulate calcium-handling proteins such as SERCA2a and modulate β-adrenergic receptor density and sensitivity, thereby amplifying adrenergic responsiveness [49]. These combined mechanisms lead to higher heart rate, improved myocardial performance, and increased tissue oxygen delivery in order to sustain enhanced metabolic demands [50,51].

Studies analyzing the interactions between THs and cardiac physiology in cohorts of healthy patients are limited. Among these, a prospective study examined a number of parameters of cardiological and endocrinological interest to determine how thyroid hormones, specifically FT4 and TSH, affected cardiovascular function in preschool-aged children [8]. TSH and FT4 levels, left ventricular (LV) mass by echocardiography, arterial stiffness by carotid–femoral pulse wave velocity (CFPWV), and systolic and diastolic blood pressure were measured in a group of 4251 children, all of whom had an average age of 6 years. Additionally, dual-energy X-ray absorptiometry (DEXA) was used to analyze body composition. The results showed an inverse association between FT4 and LV mass, as well as between FT4 and lean mass. About 55% of the effect of FT4 on LV mass was mediated by lean mass, indicating that FT4 indirectly influences cardiac structure through modulation of body composition. TSH was inversely associated with LV mass, particularly in males, and positively correlated with systolic and diastolic blood pressure [52]. These data suggest that elevated FT4 levels correlate with a reduction in LV mass and that this effect is mediated in large part by the action of hormones on lean mass composition. The differing associations of TSH and FT4 with cardiovascular parameters show different mechanisms by which THs rule cardiovascular function in childhood, highlighting the importance of the thyroid axis as an early regulator of cardiac and vascular physiology with possible implications for adult cardiovascular disease prevention [53].

The role of TH in cardiac physiology is also confirmed in “subclinical” conditions. Banu Rupani et al. [54] examined the effects of six-month treatment with levothyroxine on specific echocardiographic parameters in a cohort of 30 pediatric patients diagnosed with subclinical hypothyroidism. The parameters evaluated were: ejection fraction (EF), fractional shortening (FS), myocardial performance index (MPI), left ventricular end-diastolic diameter (LVEDD), and left ventricular end-systolic diameter (LVESD). The group consisted of 19 males (63.3%) and 11 females (36.7%), with a mean age of 6.60 ± 2.13 years. After treatment, some statistically significant changes were observed, including: the mean EF increased significantly from 63.13 ± 3.01% to 69.07 ± 4.50% (*p* < 0.001), and the FS increased from 31.83 ± 1.62% to 35.10 ± 1.13% (*p* < 0.001). MPI also showed a significant increase, from 0.28 ± 0.02 to 0.33 ± 0.03 (*p* < 0.001). LVEDD showed a significant reduction from 3.47 ± 0.46 cm to 3.05 ± 0.40 cm (*p* < 0.001). E/E′ and LVESD did not show statistically significant changes [54]. Overall, the results seem to suggest that levothyroxine therapy may lead to improvements in cardiac function in children with subclinical hypothyroidism.

Finally, the interaction between THs and cardiac function was also supported by data on the influence of THs on prognostic impacts of thyroid hormones on outcomes of cardiac pediatric patients [55,56] and severe illness [57,58] and the beneficial effects of thyroid hormone supplementation [57].

#### 4.2.2. Cardiovascular Control via the Hypothalamic–Pituitary–Adrenal (HPA) Axis

The range of action of the HPA axis is greatly expanded by their numerous peripheral targets, including the heart. Through a widely controlled hormonal cascade, the HPA axis, which is made up of the anterior pituitary, the adrenal cortex, and the paraventricular nucleus (PVN) of the hypothalamus, coordinates the body’s reaction to stress. Corticotropin-releasing hormone (CRH) and arginine vasopressin (AVP) are released by hypothalamic neurons in response to a stress stimulus. These neurotransmitters cause the anterior pituitary to release adrenocorticotropic hormone (ACTH). The adrenal cortex then uses ACTH to promote the production and release of glucocorticoids, mainly cortisol [9].

CRH influences cardiovascular physiology both directly and indirectly. Indirect effects are mediated by the release of cortisol. Direct effects occur through receptors on cardiomyocytes and vascular walls (CRH-R2/CRH-R1): the CRHR2 receptor, predominantly expressed in the heart, mediates chronotropic and positive inotropic effects through activation of the cAMP signaling pathway [59]. The hormone–receptor interaction can trigger the release of peptides such as ANP and BNP. In addition, binding to CRH-R2 may promote an increase in coronary blood flow and directly participate in processes involved in cardiac remodeling, i.e., the structural changes that the heart enacts in response to chronic conditions such as hypertension or heart failure [9]. Furthermore, in other studies in animal models, it can be observed that central stimulation of the CRH system appears to increase blood pressure through CRHR1, while peripheral stimulation via CRHR2 tends to reduce blood pressure [59].

AVP regulates water balance and vascular tone through three receptors (V1aR, V1bR, V2R), with effects varying by tissue oxygenation and vascular region. ACTH may also directly influence vascular tone through MC2R on endothelial cells, independently of cortisol [9].

ACTH influences cardiovascular physiology mainly through the production of cortisol but can also have direct vascular effects. ACTH, in fact, acts directly on cardiovascular physiology by binding to MC2R receptors expressed on vascular endothelial cells. This interaction induces the local production of vasoactive mediators, modulating vascular tone and contributing to the regulation of blood pressure independently of cortisol via an autocrine/paracrine mechanism [9].

As already noted, each component of the HPA axis exerts direct effects on the cardiovascular system, however, it is undisputed that cortisol synthesis represents the most incisive of the final events put into play by the axis in the cardiovascular system.

Glucocorticoids influence cardiovascular physiology through genomic and non-genomic mechanisms. The effects defined as genomic are expressed through the activation of intracellular receptors. GR-mediated signaling is crucial for the maintenance of contractility and for the structural and functional organization of the sarcomere: it is closely linked to the expression and transcription of numerous genes involved in the encoding of contractile proteins and channel proteins responsible for the exchange of calcium ions between the cytoplasmic environment and the sarcoplasmic reticulum and in other key processes such as the inflammatory response [59]. By triggering intracellular signaling pathways like those mediated by PI3K and MAPK kinases, glucocorticoids can more quickly start non-genomic actions and enable instantaneous cardiac function adaptation to acute stimuli [59].

Additionally to GR, glucocorticoids can also activate mineralocorticoid receptors (MRs) in cardiac tissue [60]. This is made possible by the heart’s low expression of the enzyme 11β-hydroxysteroid dehydrogenase type 2 (11β-HSD2). The previously mentioned enzyme catalyzes the conversion of physiologically active cortisol into its inactive metabolite cortisone, which has a low affinity for MRs, in mineralocorticoid-sensitive organs. In tissue environments where it is essential that glucocorticoids do not compete for MR activation, this enzyme is required. Glucocorticoids’ interaction with MR tends to promote inflammation and fibrosis, processes involved in cardiac remodeling and dysfunction [61]. At the level of the vascular endothelium, glucocorticoids appear to be responsible for and to exert protective effects under conditions of acute stress due to the anti-inflammatory mechanisms and the regulation of vascular reactivity that they activate [62]. Chronic exposure to such stimuli, however, can impair vascular repair mechanisms, promoting chronic conditions of vascular dysfunction such as hypertension. This duality underlines the complexity of endocrine–cardiovascular interactions in cardiovascular physiology and the potential consequences of HPA axis dysfunction [60].

In addition to glucocorticoids and mineralocorticoids, adrenal medullary hormones also contribute significantly to cardiovascular regulation. Catecholamines (epinephrine and norepinephrine) exert their effects primarily through the activation of adrenergic receptors on cardiomyocytes, vascular smooth muscle cells, and endothelial cells. Binding to β_1_-adrenergic receptors increases intracellular cAMP levels, leading to enhanced calcium influx, positive chronotropic and inotropic effects, and improved cardiac output. β_2_-adrenergic stimulation promotes vasodilation in skeletal muscle and coronary arteries, optimizing blood flow distribution during stress [63]. Conversely, activation of α_1_-adrenergic receptors in the peripheral vasculature increases intracellular IP_3_ and DAG, mobilizing calcium from the sarcoplasmic reticulum and causing vasoconstriction, thereby contributing to the maintenance of systemic vascular resistance and blood pressure [64]. Chronic catecholamine excess often results in sustained hypertension, concentric left ventricular hypertrophy, arrhythmias, and in some cases catecholamine-induced cardiomyopathy [65,66]. Conversely, catecholamine deficiency or secondary adrenal suppression following prolonged corticosteroid therapy impairs the cardiovascular stress response, leading to hypotension, reduced perfusion, and increased vulnerability during acute illness or surgery [67].

#### 4.2.3. Insulin’s Roles in Cardiac Metabolism and Vascular Function

With effects that differ greatly depending on physiological conditions and insulin resistance, insulin plays a crucial role in the cardiovascular system through both direct and indirect mechanisms [29].

The heart is highly dependent on the availability of metabolic substrates and requires an enormous amount of ATP to maintain contraction and electrolytic activity. Both directly and indirectly, through their effects on peripheral tissues and the heart muscle, circulating insulin levels, which vary with dietary intake and circadian rhythms, shape cardiac metabolism [29]. Cardiac mitochondria occupy a significant portion of heart cells and are responsible for producing large amounts of energy (ATP). The heart mainly uses long-chain fatty acids as fuel, followed by glucose and lactate. This flexibility in metabolism depends on a continuous oxygen supply and is essential for meeting the heart’s energy needs [68,69].

Studies on pediatric samples show a correlation between body mass and cardiac mass, suggesting that insulin modulates cardiac growth indirectly, via metabolic regulation linked to systemic nutritional status, and thus regardless of hemodynamic load [70]. This relationship is also confirmed by research conducted on adolescents suffering from anorexia nervosa, who show a significant reduction in cardiac mass that improves with nutritional recovery [71].

At the intracellular level, insulin binds to the IR, activating the PI3K/Akt pathway, with Akt1 mediating cardiac hypertrophy and normal postnatal growth. IGF-1, through its receptor and hybrid complexes with the IR, exerts similar effects on cardiac tissue, promoting cell survival and expansion [72]. Glucose uptake, mainly regulated by Akt2 via GLUT4, is crucial for cardiac metabolism [73].

The direct effects of insulin include stimulation of nitric oxide synthase (eNOS) in cells, increasing nitric oxide (NO) production, which promotes vasodilation and protects against atherosclerosis. In addition, insulin increases myocardial glucose uptake via GLUT4 and stimulates adaptive cardiomyocyte growth through mTOR signaling [74].

In insulin resistance, where PI3K/Akt signaling is disrupted while other pathways, such MAPK/ERK, remain active, the indirect effects become more evident. Activated in smooth muscle and vascular endothelial cells, this pathway generates endothelin-1 and stimulates cell proliferation, so promoting vasoconstriction and pathological vascular remodeling. This indirect mechanism is one of several maladaptive responses induced by hyperinsulinemia [74].

Among the effects mediated by hyperinsulinemia there is also the activation of the renin–angiotensin–aldosterone system (RAAS). Hyperinsulinemia activates the RAAS through various mechanisms: first, it directly stimulates renin secretion by juxtaglomerular cells and indirectly through the activity of the sympathetic nervous system [75]. Hyperinsulinism can also increase angiotensinogen synthesis, leading to higher levels of angiotensin II. Moreover, visceral obesity could be a last mechanism in charge of activating the system since it not only creates an environment of chronic inflammation and oxidative stress that prolongs system activation but also stimulates angiotensinogen synthesis. Chronic renin–angiotensin–aldosterone system (RAAS) activation promotes and aggravates congestive heart failure, systemic hypertension, and chronic kidney disease. A pro-fibrotic, pro-inflammatory, and hypertrophic environment resulting from too high levels of circulating and tissue angiotensin II (AngII) and aldosterone causes remodeling and dysfunction in renal and cardiovascular tissues [76].

Apart from the abovementioned consequences, loss of insulin control over lipolysis results in an increase in free fatty acids, which activate protein kinase C (PKC) and aggravate oxidative stress and vascular stiffness. Insulin’s immunomodulating action is also compromised in conditions of insulin resistance: this compromises its capacity to reduce pro-inflammatory molecules in macrophages such MCP-1, so encouraging inflammation and atherogenesis [77]. In general, insulin protects the cardiovascular system under physiological conditions; in insulin resistance, indirect processes favoring vascular dysfunction, inflammation, and atherosclerosis predominate.

## 5. Cardiac Endocrine Function

Cardiac endocrine activity in children is predominantly mediated by the natriuretic peptides ANP and BNP, released mainly by atrial cardiomyocytes [73,74,78].

Their expression is developmentally regulated during fetal and postnatal life, responding to mechanical stress, inflammation, and neurohormonal input. The NPPA and NPPB genes encoding ANP and BNP are regulated tightly: NPPA is expressed in early cardiac development in both atria and ventricular regions and becomes more atrial-specific over time, influenced by transcription factors GATA4, NKX2-5, and TBX5 [79]. Although NPPA and NPPB tend to be co-regulated under mechanical strain such as pressure overload, BNP expression can also be upregulated independently by inflammatory cytokines, especially IL-1β and TNF-α, as observed in myocarditis [80]. In healthy conditions, natriuretic peptide secretion predominates from the atria, while ventricular production becomes clinically significant only in pathologic conditions such as heart failure [81].

These peptides exert their effects via three known receptors. NPR-A (NPR1) activates guanylyl cyclase to promote vasodilation, natriuresis, and anti-hypertrophic effects; NPR-B (NPR2) binds CNP and is more relevant to non-cardiac functions such as bone growth; and NPR-C (NPR3) serves primarily to clear circulating peptides [5].

BNP mediates potent anti-hypertrophic, anti-fibrotic, and anti-arrhythmic actions through paracrine and autocrine mechanisms [17]. BNP-deficient animal models develop ventricular hypertrophy and fibrosis even in the absence of hypertension, underscoring its role in preventing adverse remodeling [15]. Clinically, elevated NT-pro-BNP levels predict left ventricular hypertrophy independently of overt heart failure [7], though genetic variation in ANP and NPR-A may confer stronger susceptibility to hypertrophic remodeling than BNP itself [82,83,84,85,86]. BNP deficiency accelerates pro-fibrotic signaling via pathways involving noradrenaline, angiotensin II, and TGF-β [83] and limits immunologic cell recruitment via NPR-A-mediated suppression of monocyte chemotaxis [6,84]. Clinically, higher BNP and NT-proBNP levels correlate with increased risk of atrial fibrillation, ventricular arrhythmias, and sudden cardiac death [80,81,82,83,87].

ANP and BNP also regulate blood pressure and fluid homeostasis by increasing capillary permeability and relaxing vascular smooth muscle [88]. While chronic loss of NPR-A does not significantly alter long-term blood pressure, acute endothelial deletion leads to hypertension, hypervolemia, and disrupted vascular permeability [89]. In heart failure patients, BNP infusion reduces arterial pressure and systemic resistance. Natriuresis and diuresis occur both in health and disease, but an increase in glomerular filtration rate (GFR) is seen predominantly in healthy subjects [90]. These renal effects arise from afferent arteriole dilation, efferent constriction, and reduced sodium reabsorption, though NPR-A expression seems limited to glomerular and vascular structures rather than tubules [87,88,89].

Furthermore, BNP appears to modulate the sympathetic nervous system: α-adrenergic activation enhances peptide secretion while β-adrenergic stimulation suppresses it [91,92,93,94]. ANP has been shown to inhibit sympathetic nerve activity in various vascular beds in animal models, though human data remain inconsistent [91,92,93,94,95,96,97,98]. BNP infusion at low doses reduces cardiac sympathetic activity, with higher doses needed to suppress renal sympathetic output [99]. Finally, BNP suppresses RAAS activation via direct inhibition of renin and aldosterone, amplifying its natriuretic and diuretic effects [100].

## 6. Clinical Implications of Endocrine Disease-Related Cardiovascular Effects in Fetal and Pediatric Populations

Several endocrine conditions can alter cardiac function and development in fetal and pediatric age groups. The main ones are diabetes mellitus, obesity, thyroid disorders, and adrenal dysfunction.

### 6.1. Cardiac Involvement in Diabetes

Diabetes can negatively impact cardiac development and function both during pregnancy and in children affected by the disease.

In the fetal stage, maternal pregestational or gestational diabetes mellitus (GDM) disrupts normal cardiac morphogenesis as early as the first trimester [101]. This occurs through mechanisms such as oxidative stress, inflammation, and mitochondrial damage, which interfere with epigenetic regulation and molecular signaling pathways essential for heart formation [101].

Numerous epidemiological studies have demonstrated a strong and consistent association between maternal diabetes, both type 1 (T1D) and type 2 (T2D), and an increased risk of CHD in offspring.

Early reports [102], and more recent large-scale investigations, such as a Canadian population-based study involving nearly 2.3 million births between 2002 and 2010, have confirmed that the risk of CHD in infants born to mothers with pregestational diabetes is approximately 4–5%, compared to 1% in the general population [103].

This risk varies according to CHD subtype. The Baltimore-Washington Infant Study found an increased risk of laterality and cardiac looping defects (heterotaxy), outflow tract anomalies, and atrioventricular and membranous ventricular septal defects but no significant increase in left/right-sided obstructive defects, muscular ventricular septal defects, atrial septal defects, or persistent ductus arteriosus [104]. Additionally, late gestational exposure to maternal diabetes has been associated with hypertrophic cardiomyopathy [105].

The Canadian study further demonstrated that type 2 diabetes in mothers was associated with the highest risk for heterotaxy and left ventricular outflow tract obstructions, while type 1 diabetes was more strongly linked to conotruncal anomalies and atrioventricular septal defects [103]. Both types of diabetes also increased the risk of other CHDs, including right ventricular outflow tract obstructions and atrial/ventricular septal defects, though to a lesser extent.

Ghouse et al. [106] investigated whether prenatal exposure to maternal diabetes was associated with subtle alterations in neonatal left ventricular structure and function. Findings showed more pronounced changes in infants born to mothers with pre-existing diabetes compared to those with GDM, suggesting that, even in the absence of overt congenital heart defects, maternal diabetes, particularly when pre-existing, can subtly influence neonatal cardiac anatomy and function.

Gireada et al. [107] used Fetal HQ, a 2D speckle-tracking echocardiography technique, to assess whether fetuses of mothers with well-controlled GDM displayed altered cardiac shape and contractility; despite adequate glycemic control, 60% of fetuses in the GDM group exhibited at least one form of contractility abnormality (global, longitudinal, or transverse) in one or both ventricles.

The association between diabetes and CHD are supported by multiple other studies, which consistently report a 3- to 5-fold higher prevalence of CHD among infants exposed to pre-existing maternal diabetes [108,109,110,111,112].

In children diagnosed with diabetes, both T1D and T2D, various forms of cardiovascular involvement have been observed. In pediatric patients with T1D, subclinical myocardial dysfunction can emerge early, often without clinical symptoms [113]. Advanced imaging techniques have revealed reduced longitudinal and circumferential strain, altered ventricular remodeling, and early signs of myocardial fibrosis [114,115]. Cardiac autonomic neuropathy is also common in children with T1D, affecting more than 30% of patients in some cohorts, and is associated with poor glycemic variability and suboptimal adherence to therapy [113].

In pediatric T2D, which is becoming increasingly prevalent, the cardiovascular risk is compounded by additional metabolic abnormalities such as obesity, dyslipidemia, and insulin resistance. Adolescents with T2D had increased left ventricular mass and evidence of diastolic dysfunction compared to normoglycemic peers [116]. These structural and functional changes often occur within a few years of diagnosis and are compounded by endothelial dysfunction and early arterial stiffness [117].

Another important aspect concerns electrocardiographic (ECG) abnormalities. As reported by Galli-Tsinopoulou A et al. [118], T1DM youths have a sixfold increased risk for QT/QTc prolongation and should have regular follow-ups for cardiac autonomic dysfunction. A QTc length prolongation during the diagnosis of diabetic ketoacidosis and ketosis has been reported by Aygün D et al. [119]. Additionally, children with T1D are at high risk of impaired ventricular depolarization and repolarization [120]. The electrophysiological changes can precede detectable structural abnormalities and may serve as early indicators of arrhythmic risk [121].

### 6.2. Cardiac Dysfunction and Childhood Obesity

Severe obesity in children and adolescents is a significant global public health concern. According to current estimates, the prevalence of at least one cardiometabolic risk factor in children with severe obesity ranges from 67% to 86%. Obesity and insulin resistance in youth are associated with early signs of cardiac dysfunction.

Burden et al. [122] in a meta-analysis shows that obese children and adolescents exhibit increased left ventricular mass and cardiac wall thickening compared to their normal-weight peers. Impairments in both diastolic and systolic function are observed, even in the absence of clinical symptoms. The risk of developing heart disease in adulthood therefore appears to be already increased in childhood.

Childhood obesity is associated with changes in myocardial geometry and function indicating early onset of unfavorable alterations of the myocardium [123]. Erbs et al. [124], using cardiac MRI, observed that left atrial enlargement aligns with the pathophysiological model whereby obesity increases blood volume, cardiac output, and peripheral vascular resistance, ultimately leading to cardiac chamber dilatation, a condition that appears, at least at this stage, to be reversible with weight reduction.

Yi LF et al. [125] showed that school-age children with obesity have impaired autonomic nerve function, presenting with reduced vagal tone, which is particularly prominent in those with dyslipidemia; the more obese the children, the lower the vagal tone, which may increase the risks of cardiovascular diseases. In 2023, a cross-sectional observational study involving 70 children with a body mass index (BMI) greater than one standard deviation above the mean for Thai children was conducted at Naresuan University Hospital. The study found that cardiac functional abnormalities in childhood obesity were significantly positively correlated with BMI and several cardiac parameters, including increased ventricular wall thickness [126]. A cross-sectional study published in 2014 registered the heart rate during ten minutes in the supine position with spontaneous breathing [127]. Cardiac autonomic control was assessed by heart rate variability showing that obese normotensive children and adolescents present impairment of cardiac autonomic control [127].

As reported by Campos JO et al. [128], school-age obesity (BMI  > 95th percentile) is associated with autonomic imbalances; in particular, obesity reduces vagal (parasympathetic) activity and relative sympathetic overdrive.

Similarly, another cross-sectional study evaluated cardiac autonomic function in obese school-age children (ages ~5–8 years) [125], showing an impaired autonomic nerve function, presenting with reduced vagal tone, which is particularly prominent in those with dyslipidemia.

The role of cortisol levels in cardiovascular regulation in children with obesity has also been hypothesized. Studies have reported that overweight or obese children exhibit significantly higher cortisone/creatinine ratios (β = 1.26, 95% CI: 1.17–1.36) compared to children of normal weight, suggesting that cortisol, although not directly, may contribute to the development of hypertension in this population, thereby influencing cardiovascular risk [129].

### 6.3. Cardiac Impairment in Thyroid Disorders

Fetal hypothyroidism is associated with intrauterine growth restriction (IUGR)**,** reduced cardiomyocyte proliferation, and impaired myocardial maturation. Affected neonates often exhibit altered chamber geometry, reduced systolic and diastolic function, and elevated resting heart rates by early childhood [5].

In congenital hypothyroidism, cardiovascular involvement is particularly concerning [130]. Thyroid hormone is required for normal cardiogenesis and fetal cardiac maturation. Infants born with untreated hypothyroidism may have cardiomegaly, reduced cardiac performance, and increased peripheral resistance [131]. Fortunately, with early neonatal screening and prompt initiation of levothyroxine therapy, these complications are largely preventable.

During the entire lifespan, the cardiovascular system is a key target of thyroid hormones, as they influence cardiac output, vascular resistance, myocardial contractility, heart rate, and autonomic function [50]. In children, disturbances in thyroid function, whether congenital or acquired, can result in significant cardiovascular consequences that may be transient or long-lasting depending on the timing, severity, and duration of the endocrine imbalance.

In hyperthyroidism, commonly due to Graves’ disease in children and adolescents, excess T3 and T4 lead to an increase in resting heart rate, systolic blood pressure, and cardiac output [132]. The increased metabolic demand and sensitization of β-adrenergic receptors result in a hyperdynamic circulatory state [132]. Children may present with palpitations, restlessness, and exertional intolerance. Physical examination often reveals a hyperkinetic precordium, bounding pulses, and systolic flow murmurs [133]. Although atrial fibrillation is rare in pediatric populations, it has been reported in adolescents and may be associated with thyrotoxic cardiomyopathy, particularly in the presence of delayed diagnosis or underlying heart disease [134].

Cardiac imaging studies, including Doppler echocardiography, have shown increased left ventricular end-diastolic diameter, ejection fraction, and cardiac index in untreated pediatric hyperthyroidism [135]. There is also evidence of left ventricular hypertrophy in some cases, which is generally reversible with medical therapy (e.g., methimazole) or after definitive treatment such as radioiodine ablation or thyroidectomy [136]. With normalization of thyroid hormone levels, most functional and structural changes regress, highlighting the reversibility of cardiac involvement in the hyperthyroid state [136].

In contrast, hypothyroidism exerts depressive effects on the cardiovascular system [137]. The bradycardia and reduced myocardial contractility observed in hypothyroid children result in a low-output state, which may be clinically silent or present with fatigue, cold intolerance, and exercise intolerance [138]. Importantly, hypothyroidism increases systemic vascular resistance, leading to diastolic hypertension, a finding that can be overlooked unless blood pressure is carefully measured [138]. A frequent finding in moderate to severe hypothyroidism is pericardial effusion, which occurs due to altered capillary permeability and accumulation of protein-rich fluid in the pericardial sac [135,136]. While often asymptomatic, large effusions can cause muffled heart sounds and, in rare cases, hemodynamic compromise [139,140,141].

Even in the subclinical forms of thyroid dysfunction, where free T4 and T3 levels remain within normal limits but TSH is elevated (subclinical hypothyroidism), early signs of cardiovascular involvement have been documented in children [142,143]. These include increased carotid intima–media thickness (CIMT), a surrogate marker of early atherosclerosis, endothelial dysfunction, and alterations in heart rate variability, suggesting autonomic imbalance [140,141]. These vascular changes, though often subclinical, may signal a predisposition to future cardiovascular disease, particularly in children with additional risk factors such as obesity, sedentary lifestyle, or family history of dyslipidemia [144,145,146].

### 6.4. Cardiac Involvement in Growth Deficiency

During intrauterine life, fetuses affected by intrauterine growth restriction (IUGR) are frequently exposed to a state of chronic hypoxia due to placental insufficiency. This sustained low-oxygen environment has a profound impact on fetal development, particularly by downregulating the synthesis of IGF-I [46]. Low IGF signaling hinders cardiomyocyte proliferation, resulting in cardiomyocyte deficiency, myocardial thinning, and reduced stroke volume. Early and persistent alterations of myocardial structure and decreased cardiac function detected by echocardiography in fetal, neonatal, and juvenile patients with IUGR are reported [147]. Postnatally, these hearts are metabolically compromised, exhibit higher resting heart rates, and are at increased risk for hypertension and coronary disease [5].

During childhood, GH plays a fundamental role not only in linear growth and somatic development but also in the regulation of cardiovascular structure and function. Its biological actions are mediated both directly and through IGF-1, which exerts anabolic, metabolic, and vascular effects on various tissues, including the myocardium [48]. In children with isolated or combined GHD, several studies have highlighted the presence of cardiac alterations, even in the absence of clinical symptoms, emphasizing the importance of early recognition and monitoring of cardiovascular involvement [48].

Children with untreated GHD often exhibit a significant reduction in left ventricular (LV) mass, LV wall thickness, and end-diastolic volume when compared to age-matched healthy peers [148,149]. These findings have been consistently reported in echocardiographic and magnetic resonance imaging (MRI) studies [150]. GH and IGF-1 are known to stimulate protein synthesis in cardiomyocytes and contribute to cardiac myocyte hypertrophy during development. Their deficiency impairs myocardial growth and may lead to subclinical cardiac hypoplasia, with diminished preload and reduced myocardial compliance [48].

In addition to morphological changes, functional impairments have also been observed. Some studies have demonstrated reduced stroke volume, cardiac index, and systolic performance, particularly during exercise or stress echocardiography, indicating a decreased cardiovascular reserve [151].

Beyond myocardial structure and contractility, GH and IGF-1 also contribute to endothelial integrity and vascular tone regulation. Children with GHD often display signs of early vascular dysfunction, such as increased carotid intima–media thickness (CIMT) and reduced flow-mediated dilation, which may represent early markers of cardiovascular risk [152]. These vascular alterations are thought to be mediated by impaired nitric oxide production and increased oxidative stress in the absence of GH/IGF-1 activity [153].

Treatment with recombinant human growth hormone (rhGH) has shown favorable effects on both cardiac morphology and function. Several longitudinal studies have documented significant increases in LV mass index, improvements in ejection fraction, and normalization of diastolic parameters following GH therapy, typically within 6–12 months of initiation [148,149]. Moreover, GH replacement appears to enhance exercise capacity, likely due to improvements in myocardial contractility and oxygen delivery. These effects are most pronounced when treatment is started early in childhood, highlighting the importance of prompt diagnosis and intervention.

### 6.5. Cardiovascular Effects of Adrenal Gland Disorders

Both excessive and insufficient exposure to fetal glucocorticoids, whether due to maternal stress or antenatal corticosteroid therapy, can disrupt crucial steps in cardiac maturation. Prolonged maternal cortisol elevation induces fetal cardiomyocyte proliferation but also cardiomyocyte hypertrophy, disrupted myocardial architecture, mitochondrial dysfunction, and arrhythmias, resulting in increased perinatal mortality [44,45]. Postnatally, fetuses exposed to high levels of cortisol exhibit delayed heart rate acceleration, ECG changes, and impaired blood pressure regulation, suggesting failure to adapt to the hemodynamic stress of birth [45].

Excessive aldosterone production (hyperaldosteronism) is associated with an increased risk of atrial fibrillation (AF), as aldosterone promotes atrial enlargement and fibrosis through mechanisms independent of blood pressure [154,155]. This occurs via activation of mineralocorticoid receptors on cardiomyocytes and macrophages, increasing oxidative stress and promoting the transformation of fibroblasts into myofibroblasts [156]. Activation of the renin–angiotensin system (RAS) is also a risk factor for AF [157]. Angiotensin II induces atrial remodeling, fibrosis, and prolongation of P-wave duration, thereby increasing susceptibility to atrial fibrillation [157].

Similarly, androgens exert important regulatory effects on cardiac contractility. In patients with gonadal disorders, impaired cardiac function has been documented, with significant improvement following androgen administration. This effect has been particularly well described in Duchenne muscular dystrophy [158] but more generally applies to pediatric patients with delayed puberty, where androgen deficiency may compromise myocardial performance [159].

With regard to catecholamines, in the fetus, epinephrine and norepinephrine sustain heart rate, myocardial contractility, and vascular tone, ensuring adequate perinatal adaptation; deficiency (e.g., impaired adrenal development or defects in biosynthetic enzymes) may cause hypotension and poor cardiac output, whereas excess (e.g., maternal stress, placental dysfunction) predisposes to tachycardia, arrhythmias, and oxygen imbalance [160,161,162]. In pediatric age groups, catecholamine excess due to pheochromocytoma or paraganglioma leads to hypertension, ventricular hypertrophy, and arrhythmias, while insufficiency in the context of adrenal failure or iatrogenic suppression results in inadequate stress response, hypotension, and impaired cardiac performance [66,163].

## 7. Natriuretic Peptides as Clinical Biomarkers in Pediatric Disorders

### 7.1. BNP and NT-ProBNP in Pediatric Heart Failure

Heart failure (HF) in children is a condition with unique clinical and physiological characteristics, distinct from adult HF. It is defined as a clinical and pathophysiological syndrome resulting from ventricular dysfunction, volume or pressure overload, or a combination of both [164].

Pediatric HF is further complicated by its heterogenous etiology, clinical presentation, and compensatory mechanisms which are mainly age-dependent. Most importantly, unlike adults, where HF is often due to acquired myocardial disease, CHD represents the main cause of HF in children and especially in newborn patients. A pediatric classification for HF was proposed by Ross et al., which includes age-adapted parameters, such as feeding difficulties, failure to thrive, and exercise intolerance [165].

More recently, a more detailed scoring system designed for both children and adolescents was proposed by Connoly et al. [166], the New York University Pediatric Heart Failure Index. Unlike the Ross classification, the NYU-PHFI incorporates symptoms, necessity of medical therapy, and anatomical features such as the presence of single ventricle lesions.

Amid these clinical challenges, NT-proBNP has emerged as a diagnostic and prognostic biomarker. However, it is not yet included in the HF pediatric guidelines, due to the complex interpretation in neonates and infants. Indeed, one of the greatest challenges to overcome is the physiological pattern of BNP levels during the perinatal stage, with stability only weeks after birth [167].

In children with CHD, BNP and NT-proBNP have been extensively studied. NPs have been demonstrated to be reliable indicators of volume overload and their levels correlate with shunt and ventricular dilation [168].

Data also suggest a correlation between BNP levels, right ventricular dilation, and the severity of pulmonary regurgitation in patients with tetralogy of Fallot (TOF) [169]. Interestingly, in TOF, BNP levels also correlate with IP severity and RV end-diastolic volume, indicating that BNP may serve as a sensitive marker of RV stress and volume overload in certain CHD phenotypes [170].

NT-proBNP has also been investigated as a monitoring tool after surgery for CHD and, regardless of the presence of ventricular dysfunction, high blood levels of NT-proBNP were able to identify low cardiac output syndrome, prolonged mechanical ventilation, and ICU stay [171]. Additional studies have confirmed these findings [172], confirming that monitoring NT-proBNP levels in the perioperative stage can aid in early identification of complications.

Aside from CHD, most cases of HF in children are caused by cardiomyopathies. Periodic measurement of NPs is used in practice despite actual guideline recommendations. In is to be noted that, in the context of cardiomyopathies, some studies have identified that NPs are also a strong predictor of long-term outcome [173]. Also, a recent large multicenter registry analysis conducted by Schmitt et al. found that children with higher NP levels had a higher risk of death or heart transplant, with NT-proBNP showing a good performance in the stratification of high-risk patients [174].

### 7.2. Atrial Natriuretic Peptide in Heart Failure

ANP plays a critical regulatory role in HF, promoting vasodilation, natriuresis, diuresis, and inhibition of the renin–angiotensin–aldosterone system (RAAS) [175].

Although data on the role of ANP in pediatric patients are limited, existing studies consistently show that ANP levels are elevated in children with symptomatic heart failure compared to asymptomatic patients or healthy controls. Agnoletti et al. [176] reported mean plasma ANP levels of 232.5 ± 82.9 pg/mL in symptomatic children, compared to 48.4 ± 29.4 pg/mL in asymptomatic patients. This trend has also been observed in children with CHD [169,170].

Furthermore, ANP concentrations have also been shown to increase progressively with HF severity, showing consistency as a marker of severity in children with HF [177,178,179].

Patients diagnosed with cardiomyopathies exhibit the highest ANP levels among the reviewed studies, with median values reaching 431 pg/mL. This may reflect both increased peptide synthesis and reduced receptor sensitivity [180]. In contrast, patients with right ventricular disease show lower ANP levels, while those with congenital heart disease (CHD) display intermediate values, varying according to the anatomical defect and the significance of the shunt [181].

ANP has also attracted renewed interest in the context of pharmacological treatment of HF, specifically the use of neprilysin inhibitors such as sacubitril/valsartan. Neprilysin preferentially degrades ANP over BNP, so ANP plasma levels may provide a more dynamic reflection of therapeutic effects, an especially relevant consideration in pediatric trials such as PANORAMA-HF [182].

### 7.3. Brain Natriuretic Peptides in Pulmonary Hypertension

NPs have a central role as biomarkers in the diagnosis, risk assessment, and monitoring of pulmonary arterial hypertension. In fact, as already noted, NPs are reliable indicators of RV dysfunction, a hallmark of disease progression in PH. BNP and NT-proBNP can be used, together with other tools, to stratify disease severity and prognosis. Whereas low circulating levels are associated with low risk profile, elevated levels signal higher risk and clinical worsening [183]. BNP and NT-proBNP are also increasingly used in pediatric patients and they have been particularly investigated in NICU infants. In the work of Cuna et al., among 118 cases, BNP demonstrated a 100% sensitivity for detecting PH, thereafter confirmed by echocardiography [184]. A recent systematic review confirmed NPs have adequate diagnostic accuracy for PH and could be used to discriminate patients who should receive an echocardiogram [185]. An interesting study of Dasgupta et al. was conducted in order to evaluate NT-proBNP as a non-invasive marker for PH in children of different ages, comparing it against cardiac catheterization and echocardiography. NT-proBNP levels were significantly higher in the PH group and it correlated positively with pulmonary artery pressure and vascular resistance and negatively with pulmonary artery acceleration time and right ventricular function parameters [186].

Furthermore, many data have also established the role of BNP and NT-pro BNP as accurate markers of prognostic relevance in children. The SAUDIPH registry identified BNP as a significant marker in PH, showing that elevated BNP levels at diagnosis were independently associated with higher 1 year mortality. Therefore, data support the routine use of BNP at baseline and during follow-up to help identify high-risk patients who may benefit from intensified management strategies [187].

### 7.4. BNP in Inflammatory Syndromes

Kawasaki disease (KD) is vasculitis affecting primarily infants and is likely influenced by genetic predisposition and immune responses to infectious triggers and the most frequent cardiac complication is the development of coronary artery aneurysms (CAAs) [16]. Several studies explored BNP’s predictive value for coronary lesions in KD and a recent meta-analysis supported the role of NPs as a diagnostic tool for artery lesions [188].

MIS-C is a hyperinflammatory response following mainly SARS-CoV-2 infection. While CAA is less common in MIS-C, HF is more common [52]. NPs are usually elevated in MIS-C and can assist in identifying myocardial involvement. In a large multicenter cohort, Ludwikowska et al. reported elevated NP values even in patients without reduced LVEF or echocardiographic abnormalities [18].

NT-proBNP levels are frequently elevated in pediatric patients with sepsis or septic shock, even in the absence of structural heart disease [18]. Age-adjusted NT-proBNP cut-offs have been proposed in order to improve diagnostic capacity. Sun et al. suggest a threshold of 5000 ng/L in infants and 3800 ng/L in adolescents for mortality prediction [189].

## 8. Limitations

This narrative review, while providing a broad and integrative overview of endocrine regulation during cardiac development and pediatric heart disease, has inherent limitations linked to its methodology. Narrative reviews do not follow systematic protocols for literature selection or data synthesis, which introduces potential selection bias and limits the reproducibility of the findings.

The existing literature on the endocrine regulation of cardiac development and pediatric heart disease, while rich in experimental insights, presents several notable limitations that hinder a comprehensive understanding of this complex interplay. First, most available data derive from animal models or in vitro systems, which, although informative, may not fully recapitulate the intricacies of human fetal and neonatal physiology. Translational gaps remain significant, particularly in the hormonal regulation of cardiomyocyte proliferation, maturation, and structural remodeling.

Secondly, longitudinal human studies are scarce. Most clinical data focus on postnatal hormone levels or cross-sectional assessments, limiting our ability to understand causal relationships between prenatal endocrine disruptions and later cardiovascular outcomes. Moreover, few studies have evaluated the long-term effects of altered fetal hormonal environments (e.g., maternal hypothyroidism, glucocorticoid overexposure) on cardiac function across the lifespan.

Another major limitation is the lack of standardized reference values for key endocrine biomarkers in neonates and children. Hormones such as BNP, NT-proBNP, IGF-I, and T3/T4 vary considerably with age, sex, and developmental stage, yet age-specific norms are often underreported or inconsistently applied across studies. This hampers clinical interpretation and limits the development of reliable diagnostic or prognostic tools in pediatrics.

Furthermore, many studies fail to account for biological variability, including sex differences, epigenetic regulation, and the influence of maternal–fetal factors (e.g., placental hormone metabolism, maternal diabetes, or obesity). These elements may significantly affect cardiac–endocrine interactions but are often not stratified or adequately addressed in available data. In addition, the interpretation of hormonal biomarkers in children is inherently limited by physiological variability across developmental stages. Age, sex, and pubertal status strongly influence circulating levels of TSH, IGF-I, sex steroids, and natriuretic peptides, making it difficult to establish universal thresholds. These developmental dynamics highlight the need for age- and sex-specific reference ranges when applying hormonal biomarkers in pediatric cardiovascular risk assessment.

Finally, there is limited exploration of multihormonal interactions. Most studies investigate single hormones in isolation, whereas fetal and postnatal cardiac physiology is regulated by a highly integrated hormonal network. Understanding how thyroid hormones, glucocorticoids, insulin, and growth factors interact synergistically or antagonistically remains an important but underexplored area of research.

To address these gaps, future studies should prioritize prospective, multicenter human cohorts, integrate multiomics and systems biology approaches, and aim for standardization of biomarker thresholds across developmental stages. Only through such efforts can the field advance toward personalized and preventive strategies in pediatric cardiovascular health.

## 9. Conclusions

Hormones regulate cardiac function through complex endocrine, paracrine, and autocrine pathways, influencing heart rate, myocardial contractility, vascular tone, and overall cardiac remodeling. Importantly, the heart is not only a passive target of hormonal regulation but also functions as an active endocrine organ, capable of synthesizing and secreting bioactive substances such as natriuretic peptides. These cardiokines contribute to the local and systemic modulation of cardiovascular homeostasis, thus playing a role in the autonomous regulation of cardiac function.

The synergistic interaction among hormonal signals, together with the maintenance of physiological homeostasis, is fundamental to ensure proper cardiac development and function, especially during growth. Early detection and targeted hormonal management during pregnancy are critical to mitigate these risks and support lifelong heart health. In pediatric populations, understanding the role of endocrine mechanisms in cardiac physiology is crucial, as alterations in hormonal signaling may lead to early-onset structural or functional heart abnormalities. Such dysregulations may act as silent precursors of cardiovascular disease later in life.

In clinical practice, selected hormonal biomarkers may provide valuable support for pediatric cardiovascular screening and follow-up. Table 2 summarizes the practical application of key hormonal biomarkers in pediatric cardiovascular medicine, highlighting their role in both screening (initial evaluation and risk identification) and follow-up (disease monitoring and management).

Gaining deeper insight into the molecular mechanisms that mediate hormone–heart crosstalk, as well as identifying age-specific patterns of cardiac endocrine response, may pave the way for the development of targeted preventive and therapeutic interventions. Ultimately, a comprehensive approach to the endocrine regulation of the heart in children will enhance early diagnosis, individualized care, and long-term cardiovascular outcomes in pediatric patients.

## Figures and Tables

**Table 1:** Major hormones involved in cardiovascular development and function, with their roles and effects of dysfunction during fetal life.

Hormone	Main Cardiovascular Functions	Effects of Dysfunction During Fetal Development
Thyroid hormones	Promote cardiomyocyte terminal differentiation, sarcomere maturation, mitochondrial biogenesis, and metabolic switch from glycolysis to oxidative phosphorylation. Regulate calcium handling and contractile proteins.	Hypothyroidism → impaired cardiomyocyte proliferation, abnormal chamber geometry, reduced systolic/diastolic function, increased perinatal morbidity. Hyperthyroidism → tachycardia, arrhythmias, hypertrophy.
Glucocorticoids (cortisol)	Regulate cardiomyocyte maturation, stimulate local T3 production, support IGF-II → IGF-I switch, enhance calcium handling and contractility.	Excess → hypertrophy, disrupted architecture, arrhythmias, mitochondrial dysfunction. Deficit → delayed maturation, poor adaptation to birth hemodynamic stress.
Mineralocorticoids (aldosterone)	Modulate vascular tone and fluid balance via mineralocorticoid receptors.	Hyperaldosteronism → atrial enlargement, fibrosis, oxidative stress, ↑ increased atrial fibrillation risk.
Catecholamines	Essential for fetal cardiac contractility, heart rate regulation, and stress adaptation; support vascular tone and placental circulation.	Deficiency (e.g., impaired adrenal development) → reduced cardiac output, hypotension, poor stress response. Excess → tachycardia, arrhythmias, myocardial oxygen imbalance.
Growth hormone	Direct myocardial effects (↑ mass, geometry, contractility). Indirect effects via IGF-I production.	Growth hormone deficiency → reduced left ventricular mass, impaired contractility, endothelial dysfunction.
Insulin-like growth factors	IGF-II drives early cardiomyocyte proliferation; IGF-I supports hypertrophy and maturation near term; regulate PI3K/AKT/mTOR and MAPK/ERK signaling.	Low IGF signaling (e.g., intrauterine growth retardation) → reduced cardiomyocyte number, myocardial thinning, impaired growth and function.
Insulin	Supports myocardial growth, enhances glucose uptake and metabolic efficiency, promotes adaptive hypertrophy.	Insulin deficiency/resistance → metabolic inefficiency, risk of cardiomyopathy, association with maternal diabetes-related congenital heart diseases (CHDs).
Sex hormones (estrogens, androgens)	Estrogens: mitochondrial gene expression, oxidative metabolism, cardioprotection. Androgens: improve contractility, influence electrophysiological maturation.	Estrogen deficiency → impaired maturation, metabolic dysfunction. Androgen deficiency (e.g., delayed puberty) → impaired myocardial performance.
Natriuretic peptides	Regulate blood pressure, natriuresis, anti-hypertrophic and anti-fibrotic effects; biomarkers of stress and heart failure.	Deficiency → hypertrophy, fibrosis, increased arrhythmias; high neonatal brain natriuretic peptide predicts CHD severity or perinatal stress.

**Table 2:** Practical use of hormonal biomarkers in pediatric cardiovascular screening and follow-up.

Biomarker	Target Population/Clinical Context	Screening (Initial Evaluation/Risk Identification)	Follow-Up (Monitoring/Management)
BNP/NT-proBNP	Children with congenital heart disease, cardiomyopathies, unexplained, heart failure (HF) symptoms	Rarely used for primary screening	Disease monitoring, perioperative assessment, HF follow-up
IGF-I	Children with growth hormone deficiency, intrauterine growth restriction, poor growth trajectory	Growth and cardiovascular risk assessment	Monitoring treatment response and progression
TSH, FT4	Children with congenital or acquired thyroid disorders, arrhythmias, unexplained tachycardia/bradycardia, structural congenital heart disease	Routine cardiovascular risk evaluation	Monitoring disease progression or therapy
Cortisol	Newborns with maternal stress, antenatal corticosteroid exposure, suspected adrenal insufficiency	Evaluation when adrenal dysfunction is suspected	Monitoring if abnormalities detected or therapy started
Sex hormones (testosterone, estrogens)	Adolescents with delayed puberty, hypogonadism, neuromuscular diseases (e.g., Duchenne muscular dystrophy)	Evaluation of suspected pubertal or gonadal disorders	Follow-up in confirmed endocrine or cardiovascular involvement
Catecholamines	Children with suspected adrenal dysfunction, autonomic disorders, or tumors such as pheochromocytoma/paraganglioma	Assessment in cases of unexplained hypertension, tachycardia, or stress intolerance	Monitoring disease activity, treatment response, and recurrence in catecholamine-secreting tumors

## Data Availability

Not applicable.
